# Supplementary material for: Global-scale magnetosphere convection driven by dayside magnetic reconnection
Source: Nat Commun. 2024 Jan 20;15:639. doi: 10.1038/s41467-024-44992-y (PMC10799867; doi:10.1038/s41467-024-44992-y)
Supplement: Supplementary file 1 — Supplementary Information [file 41467_2024_44992_MOESM1_ESM.pdf]

# Supplementary Information for "Global-scale Magnetosphere Convection Driven by Dayside Magnetic Reconnection"

Lei Dai[1*], Minghui Zhu[1], Yong Ren[1], Walter Gonzalez[1,2], Chi Wang[1], David Sibeck[3], Andrey Samsonov[4], Philippe Escoubet[5], Binbin Tang[1], Jiaojiao Zhang[1], Graziella Branduardi-Raymont†[4]

[1*]National Space Science Center, Chinese Academy of Sciences, Beijing, 100190,China.
[2]National Institute for Space Research (INPE), São José dos Campos, São Paulo, Brazil.
[3]Goddard Space Flight Center, NASA, Greenbelt, United States.
[4]Mullard Space Science Laboratory, University College London, Dorking, UK.
[5]European Space Research and Technology Centre, European Space Agency (ESA), Noordwijk, Netherlands.

*Corresponding author(s). E-mail(s): ldai@spaceweather.ac.cn;

†

[†]Deceased:Graziella Branduardi-Raymont

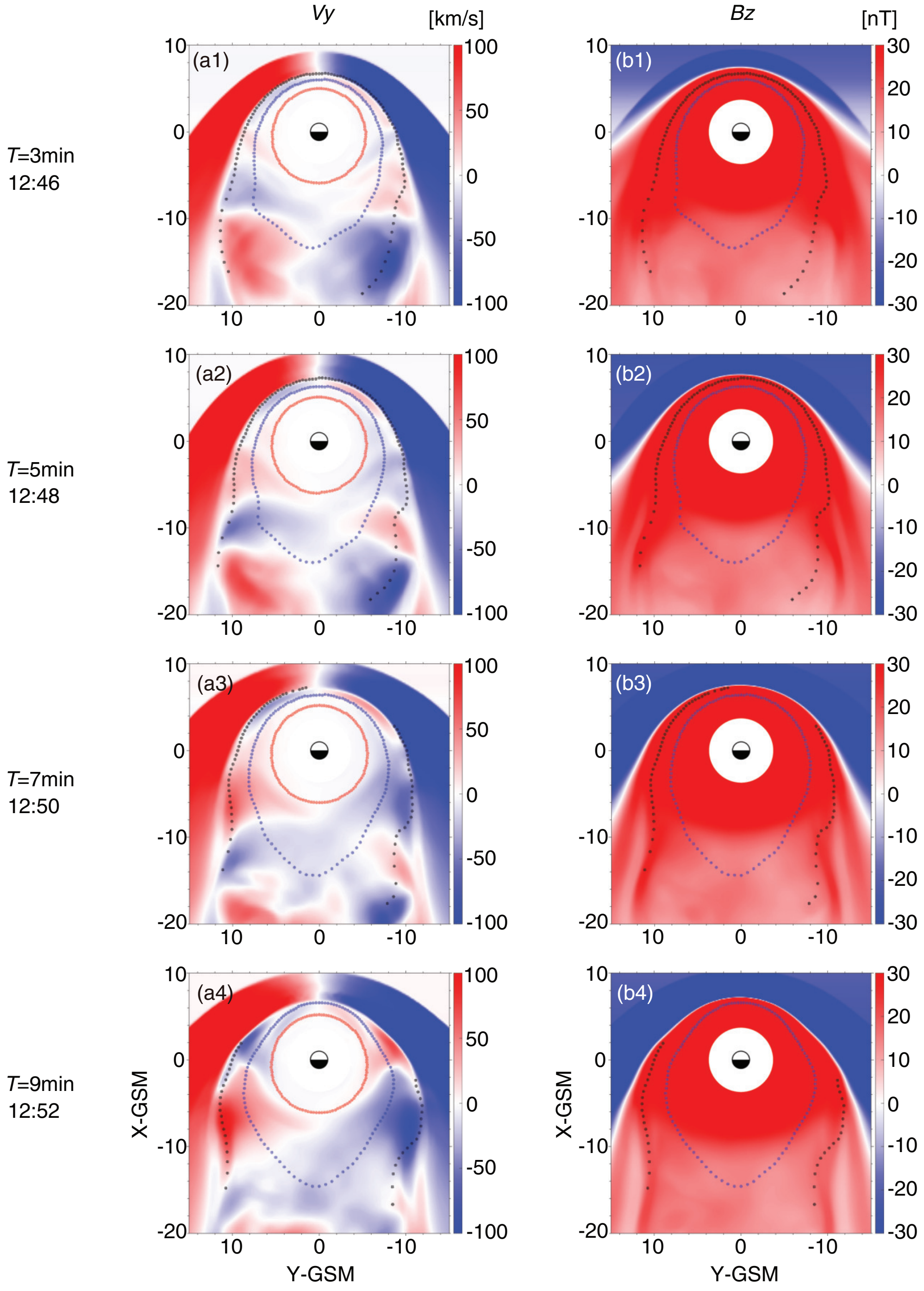


**Fig. S1** Temporal evolution of $V_y$ and $B_z$ in the XY-GSM plane in the global MHD simulations during $T$=3-9 minute.

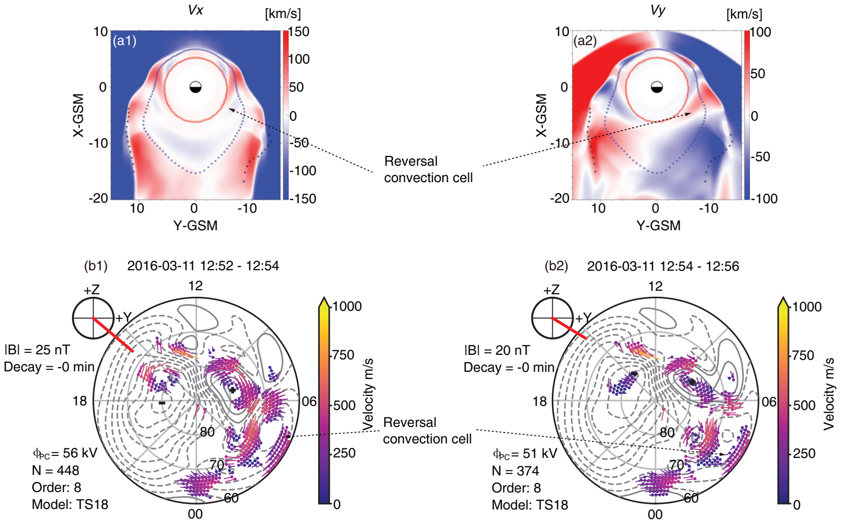


**Fig. S2** Up panel:The distribution of $V_x$, $V_y$ in the XY-GSM plane at 12:54UT. Bottom panel: ionospheric convection maps from SuperDARN during 12:52UT-12:56UT.

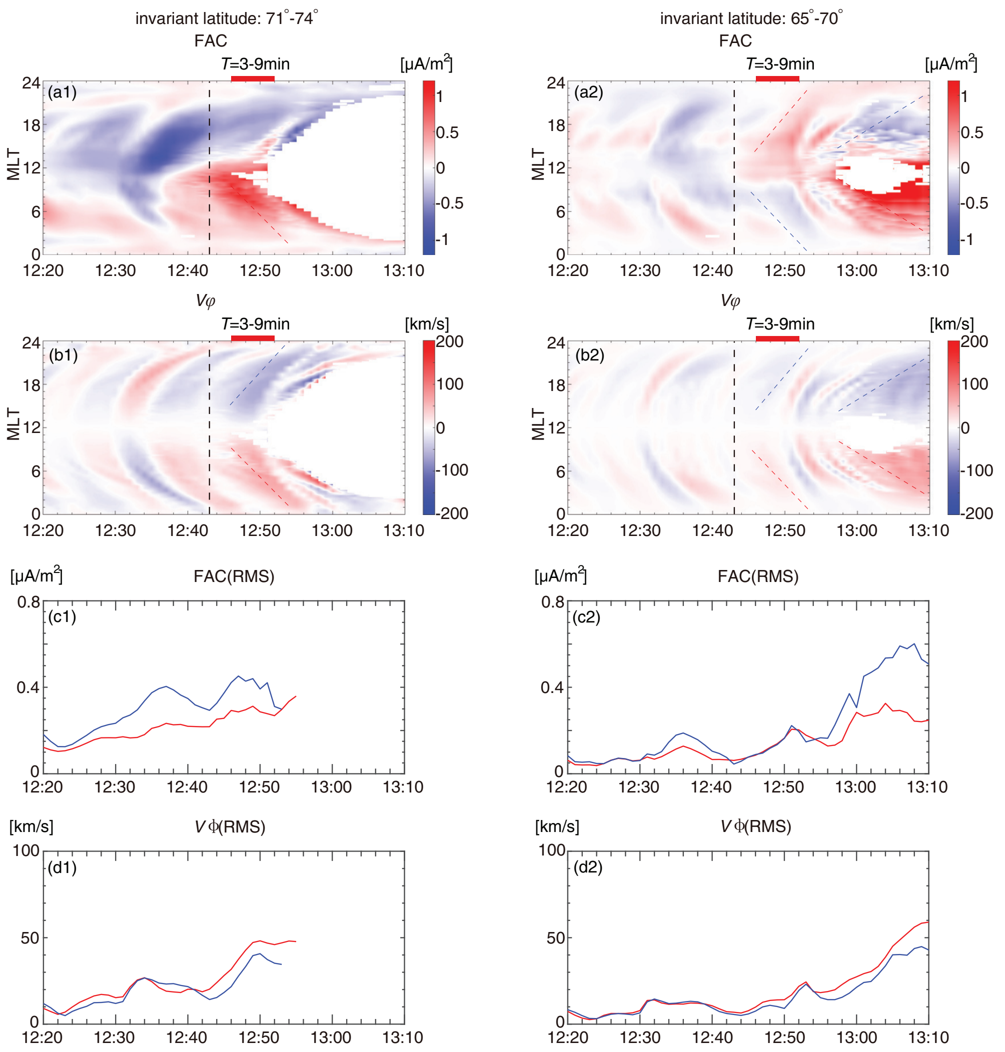


**Fig. S3** Global simulation results of FAC and $V_\phi$ with an infinite $10^8 S$ ionosphere conductivity in a test run.a1-d1) EWOgram of FAC and $V_\phi$, the root mean square of FAC and $V_\phi$ in the invariant latitude 71°-74°. a2-d2) EWOgram of FAC and $V_\phi$, the root mean square of FAC and $V_\phi$ in the invariant latitude 65°-70°.

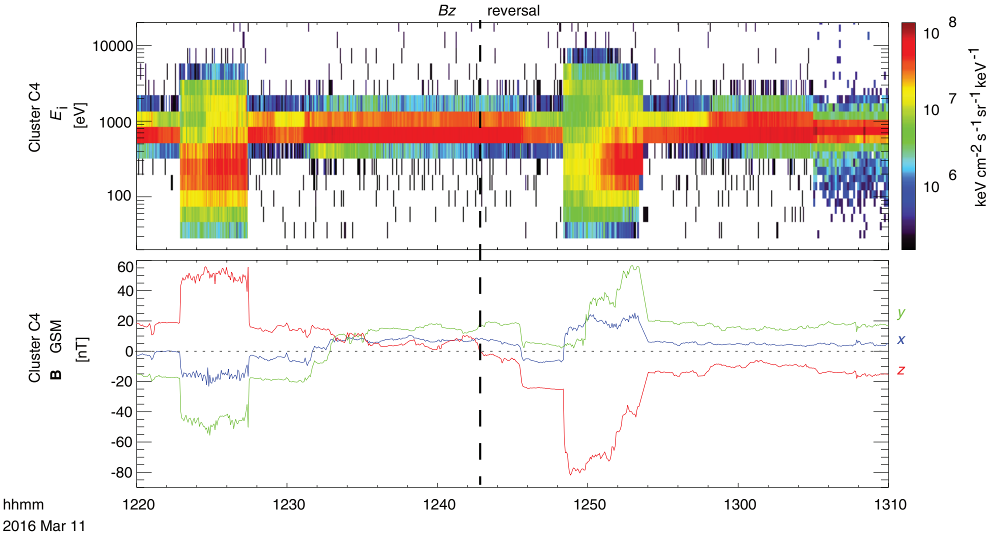


**Fig. S4** Observations from Cluster 4 near the bow shock for the event
